# Intramedullary nailing for correction of post-traumatic deformity in late-diagnosed distal radius fractures

**DOI:** 10.1007/s10195-016-0422-y

**Published:** 2016-07-26

**Authors:** Alvin Chao-Yu Chen, Chun-Ying Cheng, Ying-Chao Chou

**Affiliations:** Bone and Joint Research Center, Chang Gung Memorial Hospital-Linkou & University College of Medicine, 5th, Fu-Shin St., Kweishan Dist, Taoyuan, 333 Taiwan, ROC

**Keywords:** Distal radius, Extra-articular fracture, Malunion, Intramedullary fixation

## Abstract

**Background:**

Post-traumatic deformity of the distal radius may lead to multiple sequelae and severe functional impairment. Intramedullary fixation is a novel technique for treatment of distal radius fractures. The present study aimed to evaluate the functional and radiographic outcomes of intramedullary nailing for correction of post-traumatic deformity in late-diagnosed fractures of the distal radius.

**Materials and methods:**

From July 2009 to February 2011, 16 patients with late-diagnosed displaced fractures of the distal radius were included. Eligible inclusion was extra-articular fracture for more than 4 weeks. Surgical correction and internal fixation with intramedullary nailing was performed for treatment of ten AO type A2 and six AO type A3 fractures. All patients were followed up radiographically and clinically for an average of 20.3 months.

**Results:**

All fractures achieved bone union without major complications. Functional status and radiographic alignment significantly improved postoperatively. There was no significantly secondary displacement comparing early postoperative and final radiographic parameters. The functional results according to the Mayo wrist scoring system were good or excellent in 94 % of patients. The mean score was 83.8.

**Conclusion:**

Surgical correction and internal fixation with the intramedullary nail is a feasible and less invasive technique with few complications in the treatment of post-traumatic deformity of the distal radius.

**Level of evidence:**

IV.

## Introduction

Distal radius fractures are one of the most common upper extremity injuries, accounting for about 8–15 % of all skeletal injuries treated by orthopedic surgeons [1–3]. Late-diagnosed fractures are frequently complicated with many sequelae; among them, malunion and post-traumatic wrist arthritis due to post-traumatic distal radius deformity are most frequently seen [4–6]. Common deformities following an extra-articular distal radius fracture include loss of the normal volar tilt of the articular surface in the saggital plane, decreased ulnar inclination in the frontal plane, and loss of length relative to the ulna [6]. Post-traumatic deformity results in alteration of normal anatomy, biomechanics of distal radius, and functional impairment in hand and wrist [6–8]. Many surgical modalities in the treatment of acute fractures of the distal radius have been proposed [9, 10]. Intramedullary nailing is currently used to treat unstable extra-articular fractures of the distal radius [11]. Bearing the advantages of allowing load transfer across the fracture site and lessening soft-tissue dissection, intramedullary fixation can be used to stabilize the fracture bones through a less invasive approach and maintain periosteal vascular blood supply to promote fracture healing [12]. The purposes of this retrospective study were to describe our experience with mini-open osteotomy, local bone grafting, and fracture stabilization with the intramedullary nail device for treatment of post-traumatic deformity in late-diagnosed fractures of the distal radius. Clinical outcomes, radiographic analysis, and complications were reported.

## Materials and methods

**Fig. 1:**
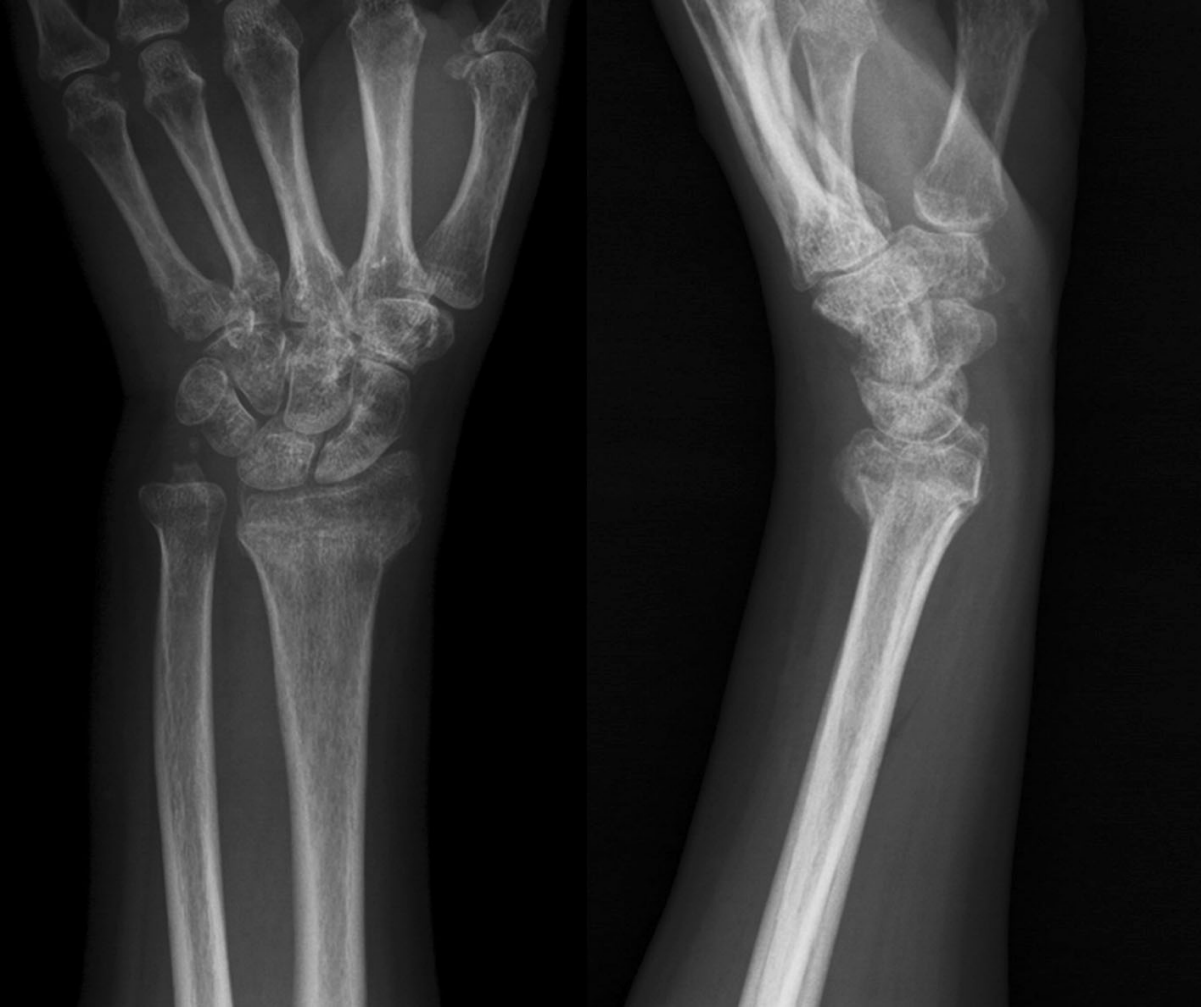
A 69-year-old female patient. Radiographs of left wrist at 6 weeks after injury show fracture of the distal radius with malalignment

**Fig. 2:**
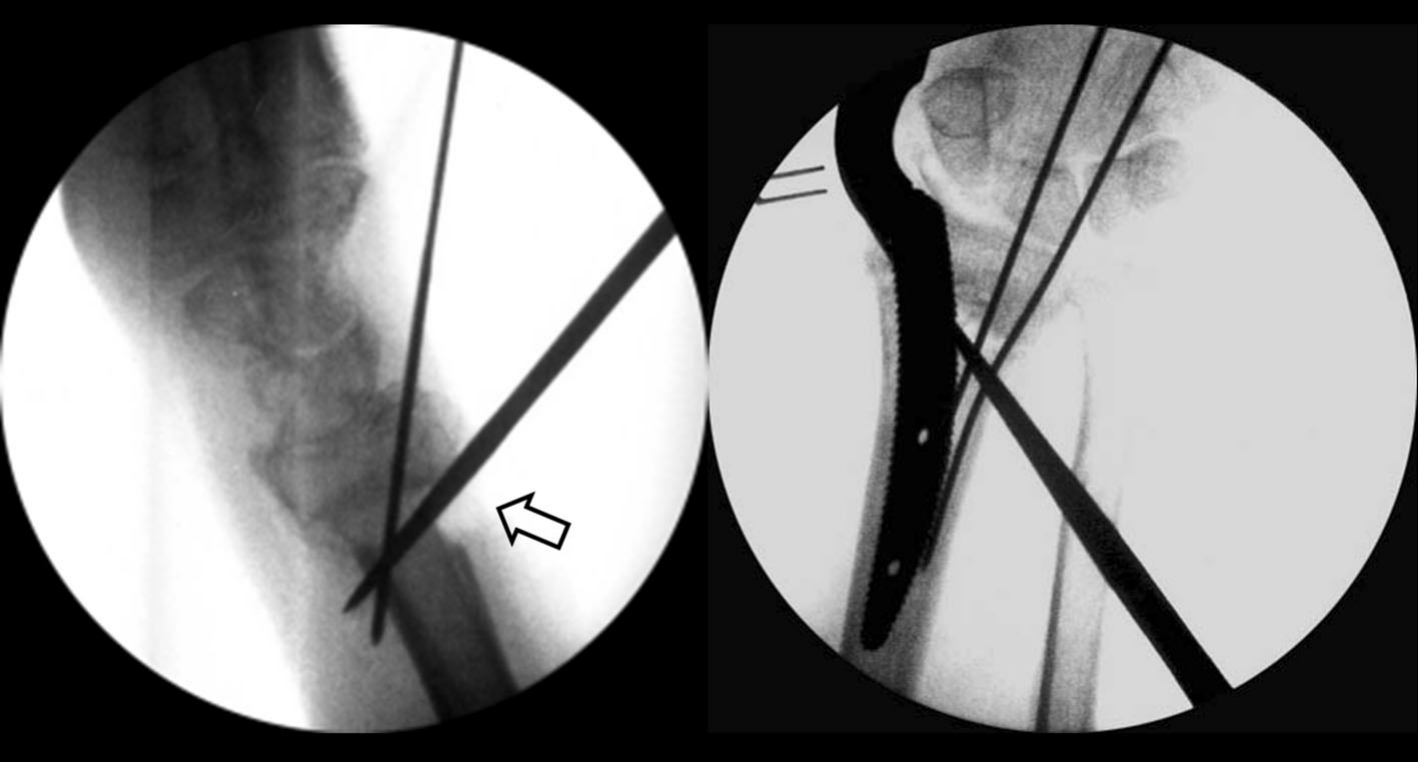
Intra-operative C-arm images show correction of deformity through mini-open osteotomy and provisional Kirschner wire fixation (*hollow arrow*), and intramedullary preparation by broach taping

In total, 16 consecutive patients (Table 1) with displaced extra-articular distal radius fractures (Fig. 1) and injured for more than 4 weeks were treated with mini-open wedge osteotomy (Fig. 2) and intramedullary fixation device (Micronail^®^, Wright Medical Technologies, Arlington, TN, USA), from July 2009 through February 2011 by a single surgeon. There were 5 men and 11 women, with an average age of 61.8 years (range 49–81 years). The fracture involved 5 right wrists and 11 left wrists in equal numbers of patients. According to the AO fracture classification [13], there were 10 AO type A2 and 6 AO type A3 fractures. The mechanisms of injury in all patients were either a simple fall on the outstretched hand or a traffic accident. The mean time from injury to surgery was 10.7 weeks (range 4–18 weeks). All patients had regular follow-up in our outpatient clinic for at least 12 months.

**Table 1:** Patient demographic data

Patient no.	Age (years)	Sex	Wrist	AO fracture classification	Time to surgery (weeks)	Bone union (weeks)	Follow-up (months)
1	65	F	Left	A2	4	12	32
2	64	M	Left	A2	16	6	28
3	69	F	Left	A3	8	8	30
4	81	M	Left	A2	6	6	24
5	58	F	Left	A2	10	8	32
6	59	F	Left	A2	7	6	12
7	49	M	Right	A3	16	6	20
8	58	F	Left	A2	9	6	18
9	71	F	Left	A3	7	8	16
10	67	M	Left	A3	4	6	16
11	49	F	Left	A2	13	7	12
12	55	F	Right	A2	18	6	16
13	62	F	Right	A3	10	7	18
14	57	M	Right	A2	14	6	14
15	66	F	Left	A2	18	12	18
16	59	F	Right	A3	11	12	18
Mean	61.8				10.7	7.6	20.3
SD	8.2				4.7	2.3	6.8

*SD* standard deviation

### Surgical procedure

A 2-cm longitudinal incision was made medial to Lister’s tubercle on the dorsal wrist. An open-wedged osteotomy was performed through the malunion site using a 5-mm osteotome, and transfixed provisionally with Kirschner wires after manual reduction under C-arm fluoroscopic assistance. A second 1-cm incision was made over the radial styloid, followed by blunt soft-tissue dissection and meticulous protection of the superficial radial sensory nerve. Dissection through the interval of the first and second dorsal extensor compartments was made using a starter awl in order to create a cortical bone window. This was followed by tapping sequential broaches into the intramedullary canal until the proper fit was achieved (Fig. 2). After sizing and trialing, a Micronail^®^ of the measured size was gently inserted through the pre-taped track into the medullary canal of the distal radius. Three distal fixed-angle locking screws and two proximal interlocking screws were then applied through the guiding system. After satisfactory realignment and secure fixation were confirmed by fluoroscopy, all provisionally transfixed Kirschner wires were removed and the guide system was disassembled from the intramedullary nail. Local callus from the nascent malunion was morselized to serve as a bone graft for the osteotomy site in 14 patients (87.5 %). Two patients (12.5 %) needed additional artificial bone graft substitutes due to insufficient local bone graft. The wound was closed layer by layer. A volar short arm splint was applied for protection after dressing the wound.

### Postoperative evaluation and follow-up

All patients had a short arm splint for temporary protection postoperatively. To avoid stiffness and reduce swelling, postoperative rehabilitation with active finger, elbow, and shoulder range of motion training was started immediately postoperatively. Patients were typically seen for their first postoperative visit at 10–14 days. At that visit, the short arm splint and sutures were removed and unrestricted active range of motion was allowed for the wrist. Posterior–anterior and lateral radiographs of the injured wrist were performed preoperatively, then monthly for 3 months, and then every 3 months until 1 year after surgery. Three radiographic parameters, radial height, radial inclination, and volar tilt, were recorded. Bone healing was evaluated and radiographic union was confirmed with trabecular bridging of fracture. The statistical analysis was a 2-sample *t* test to determine the significance of the radiographic parameters between postoperative radiographs and those of the 1-year follow-up. Significant difference was defined as a *p* value less than 0.05.

Functional evaluation was performed using the modified Mayo wrist scoring system. Grip strength of the injured side was measured and reported as a percentage of maximal strength of the contralateral side. The pain scale was self-reported and graded with the use of a questionnaire. With the addition of the satisfaction score, a modification of the Mayo Wrist Scoring Chart was used for functional assessment, allowing for a total count of 100 points in four categories [14].

## Results

**Fig. 3:**
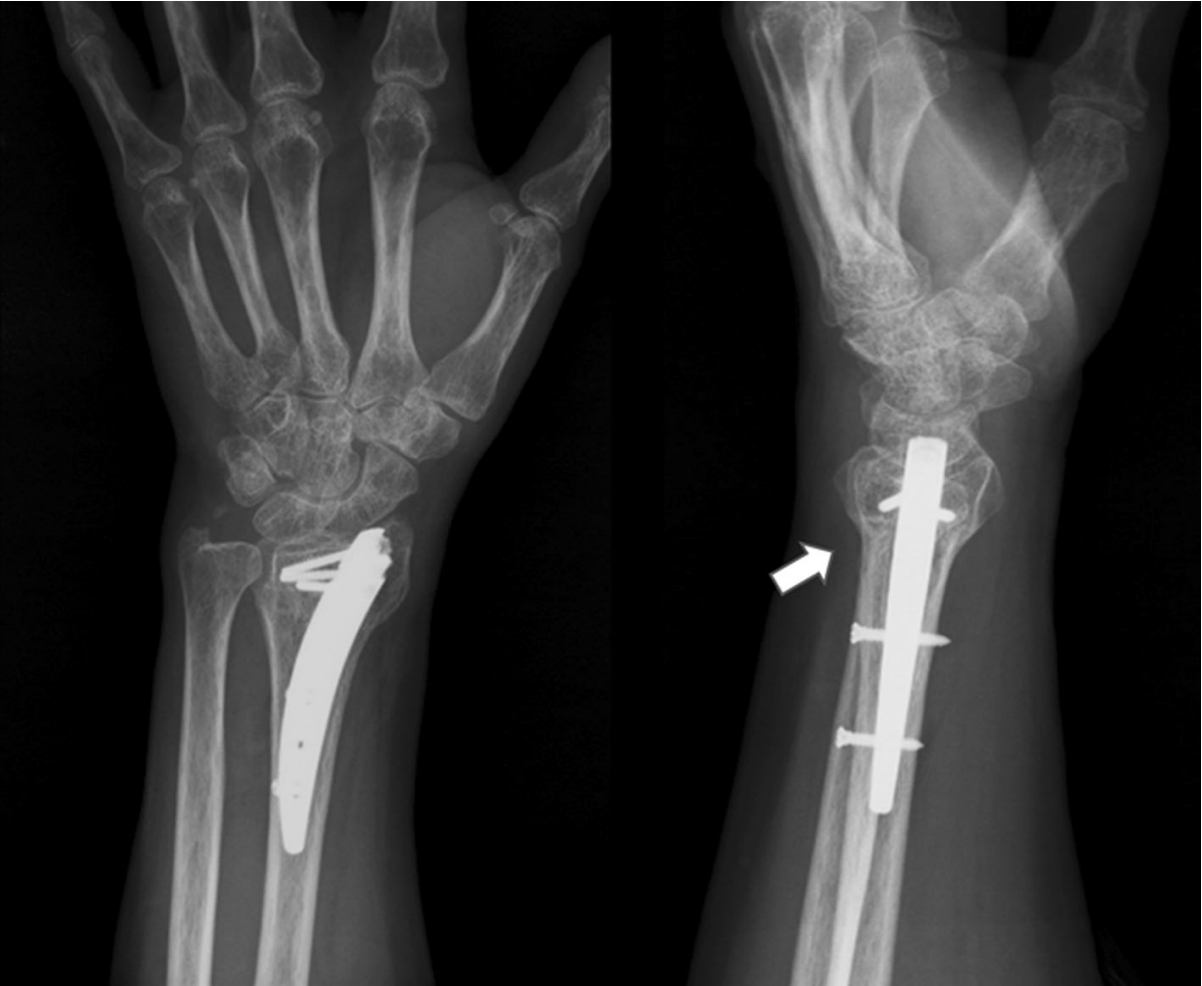
Radiographs at 6 months after surgery show osseous union and consolidation of osteotomy site (*white arrow*)

The average follow-up period was 20.3 months (range 12–32 months). All 16 patients resumed hand and wrist motion and had healed fractures (Fig. 3). The mean osseous healing time was 7.6 weeks (range 6–12 weeks) after surgery. All measurements of grip strength, range of motion, pain, and functional scores were significantly improved at the 1-year follow-up compared with preoperative status (Table 2). The overall clinical and functional results according to the modified Mayo wrist scoring system were excellent in 8 patients (50 %), good in 7 patients (44 %), and fair in 1 (6 %). All achieved satisfactory functional outcome. The mean Mayo score was improved from 41.9 (range 20–65) preoperatively to 83.8 (range 70–100) at final follow-up.

**Table 2:** Statistical summary of functional outcomes

	Grip strength (kg), mean (SD)	Range of motion (°)				Pain VAS	Functional outcome
		Extension, mean (SD)	Flexion, mean (SD)	Supination, mean (SD)	Pronation, mean (SD)		Mayo, mean (SD)
Preoperative	10.8 (2.9)	35.6 (13.4)	39.7 (9.7)	22.2 (6.6)	73.1 (8.1)	1.2 (0.7)	41.9 (14.6)
Postoperative^a^	22.6 (6.2)	71.3 (12.4)	62.6 (12.6)	76.6 (10.1)	81.9 (9.5)	0.3 (0.4)	83.8 (10.9)
*p* value	<0.001	<0.001	<0.001	<0.001	<0.001	<0.001	<0.001

*SD* standard deviation, *VAS* visual analogue scale

^a^At 1-year follow-up

All the measurement of postoperative radiographic parameters showed significant improvement compared with preoperative status (Table 3). Radial height improved from an average of 8.0 mm (range 6–12) preoperatively to 12.1 mm (range 7–19) postoperatively. Radial inclination improved from an average of 16.4° (range 3–24) preoperatively to 21.9° (range 16–30) postoperatively. Volar tilt improved from an average of 4.0° (range −30 to 11) preoperatively to 11.2° (range −10 to 11) postoperatively. No significant secondary displacement was found when comparing all the three parameters between early postoperative status and the final follow-up.

**Table 3:** Statistical summary of radiographic findings

	Radial height (mm)			Radial inclination (°)			Volar tilt (°)		
	Preop	Postop	1-year	Preop	Postop	One year	Preop	Postop	1-year
Mean	8.0	12.1	11.2	16.4	21.9	21.1	4.0	11.2	4.8
SD	2.6	3.4	10.4	5.7	3.8	2.9	4.2	3.1	3.9
*p* value^a^	0.001	0.079		0.001	0.200		<0.001	0.424	

*Preop* preoperative, *Postop* postoperative

^a^ *p* value, comparison between preop and postop status, and between postop and 1-year status

There was no major complication such as wound infection, loss of reduction, implant failure, or complex regional pain syndrome. Minor complications included four patients with transient superficial radial nerve irritation. The symptoms resolved gradually in the early follow-up period. There was no case of screw penetration into the radiocarpal or distal radioulnar joint.

## Discussion

The primary goal in treatment of post-traumatic deformity of the distal radius is to restore anatomical alignment and prevent secondary displacement. Traditionally, extra-articular osteotomy is performed to re-establish volar tilt in the saggital plane and radial inclination in the coronal plane, correct any rotational malalignment in the horizontal plane, and restore radial length [14, 15]. Several fixation modalities are available for distal radius osteotomy. Osteosynthesis with dorsal and/or volar plates needs sufficient exposure to facilitate direct visualization, accurate reduction, and secure plating fixation. Common criticisms exist regarding wide soft-tissue dissection, tendon complication including tenosynovitis or tendon rupture, and prominence of hardware with internal fixation plate. Many patients need subsequent removal of the implant [16–18]. Intramedullary nails for fixation of the distal radius are intended to minimize those complications in treatment of post-traumatic deformity of distal radius [19]. With the benefits of limited soft-tissue dissection, a low-profile implant with less risk of tendon attrition, divergent subchondral screw placement, and locked fixed-angle fixation, the intramedullary nail serves as an internal splint to provide sufficient stability, allow immediate active motion at the fracture site and acquire healing through peripheral callus remodeling [11, 20, 21].

Traditionally, open wedge osteotomy is a commonly adapted procedure in correction of distal radius deformity [22, 23]. Autogenous iliac crest cancellous or corticocancellous bone graft is used to fill the gap and provide mechanical support after open wedge osteotomy. Several problems have been identified regarding this approach, including donor site morbidity, delayed union at the bone–graft interfaces, and size mismatch between the graft and the osteotomy defect [24, 25]. In dealing with the post-traumatic deformity of distal radius in our series, osteotomy was performed through the identifiable fracture line of the nascent malunion. The local callus was morselized and served as bone graft to fulfill the osteotomy gap. Additional artificial bone graft substitutes may be used as an adjunct in case of insufficient local bone graft, and thus may decrease surgical time and donor site morbidity.

In this study, most patients (94 %) had excellent or good outcomes, which showed comparable results with other series [26–28]. One patient (6 %) did not receive any definite management until 13 weeks after injury, and showed only fair results. Inferior outcome could be attributed to soft-tissue contracture and wrist stiffness after prolonged immobilization, and delayed fracture healing after osteotomy [29]. Performing correction osteotomy with intramedullary nail fixation for post-traumatic distal radius deformity at an earlier stage may help prevent maladaptive soft-tissue contractures and arthrosis of the distal radio-ulnar joint [30].

Potential complications of intramedullary nail fixation using Micronail^®^ may include dorsal superficial radial sensory nerve injury, soft-tissue irritation with screw heads, and screw penetration into the articular surface [20, 21]. Those complications are uncommon. None of the above complications occurred in our series. None of our patients presented residual paresthesia or neurological sequelae. Normally, the superficial radial sensory nerve may be present in the surgical field and is highly susceptible to injury. It is recommended that the superficial radial sensory nerve be identified and protected throughout the surgical procedure. There were no cases of infection, complex regional pain syndrome, hardware failure, or soft-tissue irritation in our series.

The limitations of this study include the small sample size with limited patient numbers, and short-term follow-up period. Further prospective and long-term studies would be helpful to delineate the effect on outcome of the timing of stabilization. In addition, larger outcome studies with direct comparison to other osteosynthesis techniques are necessary to better evaluate the efficacy of intramedullary nail fixation in correction of post-traumatic radius deformity.

In conclusion, intramedullary nail fixation for correction of post-traumatic deformity of the distal radius is a less invasive technique which can provide satisfactory reduction and stable fixation. Surgical osteosynthesis with local callus bone grafting is a feasible and effective management option with satisfactory radiographic and functional outcomes.
